# Preoperative Splenic Density for the Prediction of Survival and Adjuvant Chemotherapy Benefits in Gastric Cancer

**DOI:** 10.7150/jca.47559

**Published:** 2020-08-25

**Authors:** Wei-teng Zhang, Ce Zhu, Xiang Wang, Su-jun Wang, Xiao-dong Chen, Xiao-lei Chen, Xian Shen

**Affiliations:** 1 Department of Gastrointestinal Surgery, The Second Affiliated Hospital, Wenzhou Medical University, Wenzhou, Zhejiang, China; 2 Department of Gastrointestinal Surgery, The First Affiliated Hospital, Wenzhou Medical University, ShangCai Village, Wenzhou, Zhejiang Province, China

**Keywords:** gastric cancer, chemotherapy, splenic density, prognostic factor

## Abstract

**Background**: We aimed to determine whether splenic features change during tumor progression by evaluating the clinicopathological characteristics relevant to splenic density in patients with gastric cancer (GC) and identify a new predictive indicator of prognosis and chemotherapy benefits.

**Methods:** In the present analysis, 408 patients who underwent gastrectomy were included. Density was expressed in mean spleen Hounsfield units on computed tomography. Other clinical characteristics and detailed follow-up data were collected. The cutoff splenic density was 47.8 by the Xtile software. The R software was used for characteristic differential analysis in patients with different splenic densities. The Cox proportional hazards model and forest plot were used for prognosis and chemotherapy benefit analyses.

**Results:** Patients with low splenic density had significantly worse 3-year disease-free survival (DFS) and overall survival (OS) rates (high vs low splenic density: DFS, 63.4% vs 44.6%, p<0.001; OS, 69.8% vs 52.4%, p<0.001). Splenic density showed strong negative correlations with age, number of metastasized lymph nodes, tumor size, and depth of tumor invasion. The benefits of adjuvant chemotherapy were better in the low splenic density group (hazard ratio of OS, 0.546; p=0.001) than in the high-density group (hazard ratio of OS, 0.701; p=0.106).

**Conclusions:** Patients with low splenic density tended to have more advanced tumors and poor prognosis, but better chemotherapy benefits. Splenic density can be regarded as a new indicator of chemotherapy benefits and increase the accuracy of preoperative staging evaluation. Moreover, preoperative evaluation of splenic density may help establish individualized treatment strategies.

## Introduction

Gastric cancer (GC) is one of the most prevalent types of malignancy worldwide 1. Compared with other malignant tumors, GCs are more advanced at diagnosis, which leads to a poorer prognosis 2. Gastrectomy with adequate lymph node (LN) dissection is the only effective choice of treatment for these patients 3. However, the prognosis after radical resection is still poor owing to the high recurrence/metastasis rate 4. Adjuvant chemotherapy was recommended for patients with advanced GCs 5, but not all patients benefit from chemotherapeutics 6-8. These findings indicate that the present tumor-node-metastasis (TNM) system provides inadequate information for prognosis and that new prognostic biomarkers are required to improve survival predictions.

LN metastasis, especially in the splenic hilar and artery, has been recognized as a sign of poor prognosis, and whether the benefits of splenic resection advance GC has been controversial recently. Some studies indicated that spleen-preserving hilar LN dissection may be more beneficial for patients 9, 10. As the largest immune organ, the spleen contains a large number of B and T lymphocytes, which play important roles in tumor immunity 11, 12. Han's study explains the reason why an enlarged spleen is associated with poor survival in advanced liver cancer 13. Ter-cells are enriched in the enlarged spleen of hosts bearing advanced tumors and facilitate tumor progression by secreting the neurotrophic factor artemin into the blood 13. However, the relationship between spleen characteristics and GC prognosis has not been examined, and whether the computed tomography (CT) features of the spleen change in advanced GC remains unknown.

The aim of this study was to determine whether splenic features change during tumor progression by evaluating the clinicopathological characteristics relevant to splenic density in patients with GC. We also compared overall survival and chemotherapy benefits among patients with different splenic features.

## Patients and Methods

### Patients

The data of patients with operable gastric cancer who underwent radical gastrectomy at the First Affiliated Hospital of Wenzhou Medical University, Zhejiang, China, between December 2009 and December 2012 were entered into a retrospective database. Inclusion criteria were as follows: (1) Pathological diagnosis of GC and underwent radical gastrectomy; (2) Patient did not undergo neoadjuvant therapy before surgery; and (3) Patient underwent contrast-enhanced abdominal CT scan within 2 weeks preoperatively. Exclusion criteria were as follows: (1) Patient underwent neoadjuvant chemotherapy or radiotherapy before surgery; (2) Image quality of abdominal CT scans was not conducive towards evaluation; (3) Patients with severe disease, distant metastasis, inconsistent postoperative pathology, and palliative operations. Finally, 408 patients who underwent gastrectomy and had available preoperative abdominal CT scans were included in the present analysis. Due to the retrospective nature of the study, informed consent was waived. The study was approved by the ethics committee of the Wenzhou Medical University First Affiliated Hospital and conformed to the tenets of the Declaration of Helsinki.

### Clinical and operative characteristics

The following clinicopathological characteristics were collected: (1) basic information, including sex, age, weight, height, previous abdominal surgery, American Society of Anesthesiologists (ASA) grade, comorbidities, laboratory results, histological type, tumor location, and TNM tumor stage (AJCC 8th edition TNM staging system); (2) operative method, including type of resection and reconstruction and laparoscopic surgeries; (3) postoperative outcomes, including postoperative complications (using the Clavien-Dindo classification) and hospitalization, and adjuvant chemotherapy suggestions; and (4) follow-up, including the primary (overall survival [OS]) and secondary end points (disease-free survival [DFS]). The patients were followed up until October 2016. The mean follow-up period was 48 months (range, 36-72 months). Follow-up was performed every 3 months for the first 2 years and every 6 months thereafter on an outpatient basis or via telephone interviews.

### Spleen characteristics on CT scan

The volume and mean Hounsfield units (HU) were calculated from the three-dimensional reconstructed image of the spleen on an abdominal plain CT scan (1.25 mm). Splenic density was expressed in HU (INFINITT PACS version 3.0.11.3 software, South Korea). In order to control the measured differences caused by spleen heterogeneity, the mean HU of the reconstructed spleen was recorded as the spleen density. Pre-analysis revealed a significant relationship between splenic density, but not volume, and GC prognosis; therefore, we further analyzed splenic density in the following study.

In addition, the cutoff splenic density was set as 47.8 HU using Xtile version 3.6.1 (http://tissuearray.org/). Patients with different splenic densities were segregated into two groups based on this value.

### Statistical Analyses

Statistical analyses were performed using the R version 3.6.0 software (http://www.R-project.org). The R packages used included “survival,” “rms,” “car,” “stringi,” “mgcv,” and “nnet,” which were utilized for variable analysis. Continuous parameters were presented as mean ± standard deviation. The Kruskal-Wallis test was used to analyze the differences in continuous variables between the groups, while the chi-square and Fisher's exact tests were used for categorical variables. Univariate analyses were performed using the Cox proportional hazards model. Significance was indicated by P values of <0.05. The R package corrplot was used to draw the correlation coefficient graph using Kendall method. The forest plot was provided by the packages ggplot2, forest, and rms.

## Results

### Patient characteristics

The patients' characteristics are shown in Table 1. Four hundred eight patients were assigned to the following groups: 202 (49.5%) to the high splenic density group and 206 (50.5%) to the low splenic density group.

Patients with low splenic densities were more likely to have a tumor of advanced stage (Table 1) and more platelet count. However, there were no significant differences in general patient characteristics such as body mass index, sex, ASA grade, comorbidity, tumor location and differentiation, postoperative hospitalization, and surgery-associated complications. The 3-year DFS and OS rates were significantly worse in patients with low splenic densities (high vs low splenic density: DFS, 63.4% vs 44.6%, p < 0.001; OS, 69.8% vs 52.4%, p < 0.001).

**Figure 1:**
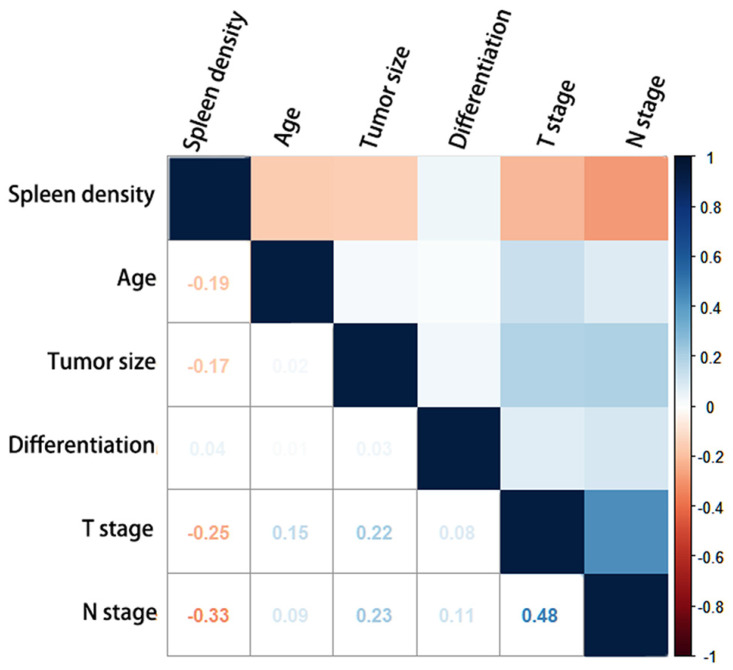
** Correlation diagram of the clinicopathological characteristics and splenic density.** The Kendall correlation coefficients between the characteristics are shown as numbers.

Figure 1 shows the correlation between splenic density and tumor characteristics. Splenic density (HU) showed strong negative correlations with age, stage of metastasized LNs, tumor size, and depth of tumor invasion. Low statistical significance was observed for tumor differentiation.

### Univariate and Multivariate Cox proportional hazards model for clinical characteristics

Patients with low splenic densities tended to have poorer DFS and OS rates (hazard ratios [HRs]: 1.56 and 1.62, respectively, p < 0.001; Table 2). Age>75 (p < 0.001), TNM stage (p < 0.001), Adjuvant chemotherapy (p < 0.001) and tumor size (p < 0.001) proved to be significant prognostic factors of OS. Besides, Patients with tumors located in the cardia showed worsened prognosis as compared with patients with tumors located in other primary regions (cardia vs antrum: HR, 1.67 vs 1, p = 0.002) but no statistically significant difference in prognosis compared to patients with tumors in other regions. Billroth I and II anastomoses were superior to Roux-en-Y in terms of overall survival (Billroth II vs Billroth I vs Roux-en-Y: HR, 0.54 vs 0.35 vs 1, p < 0.001).

Multivariate cox regression showed adjuvant chemotherapy, TNM stage and reconstruction method were independent risk factors of DFS and OS, while combined resection only shows statistical significance in DFS analysis. The splenic density was not statistically significant risk factor in the multivariate analysis due to the high correlation with TNM stage.

**Figure 2:**
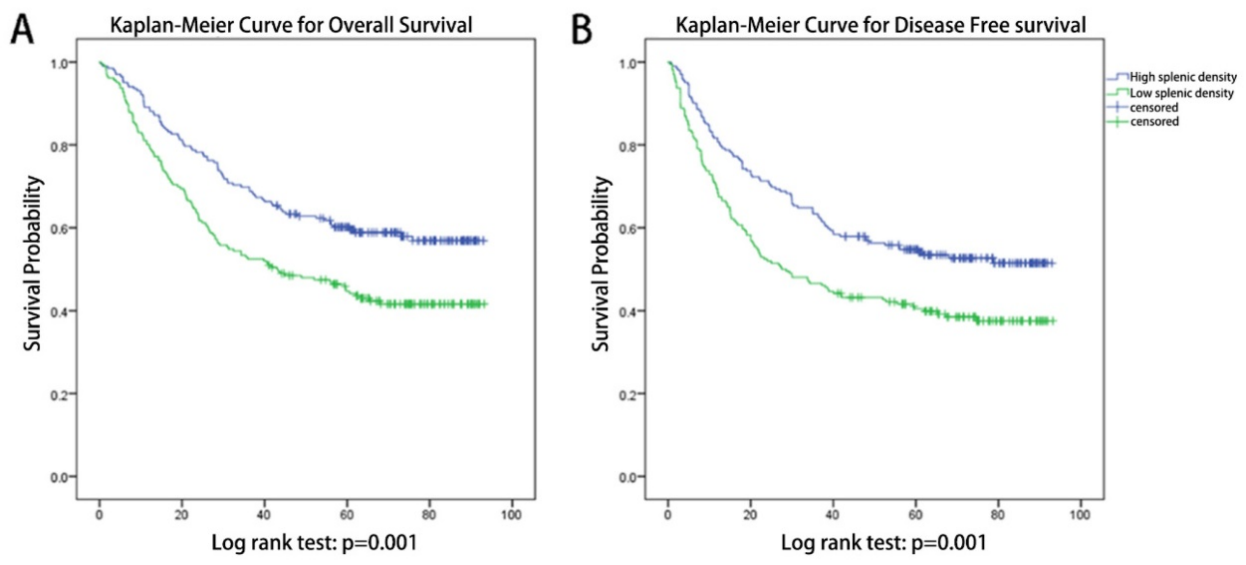
** Kaplan-Meier Curves for patients with different splenic densities.** (A) Kaplan-Meier Curves for overall survival, log-rank test: p=0.001. (B) Kaplan-Meier Curves for disease-free survival, log-rank test: p=0.001.

Kaplan-Meier Curves (Figure 2) indicated that patients with different splenic densities shows significant different prognosis, both in DFS (log-rank test: p=0.001) and OS (log-rank test: p=0.001).

### Chemotherapy benefits for patients with different splenic density

**Figure 3:**
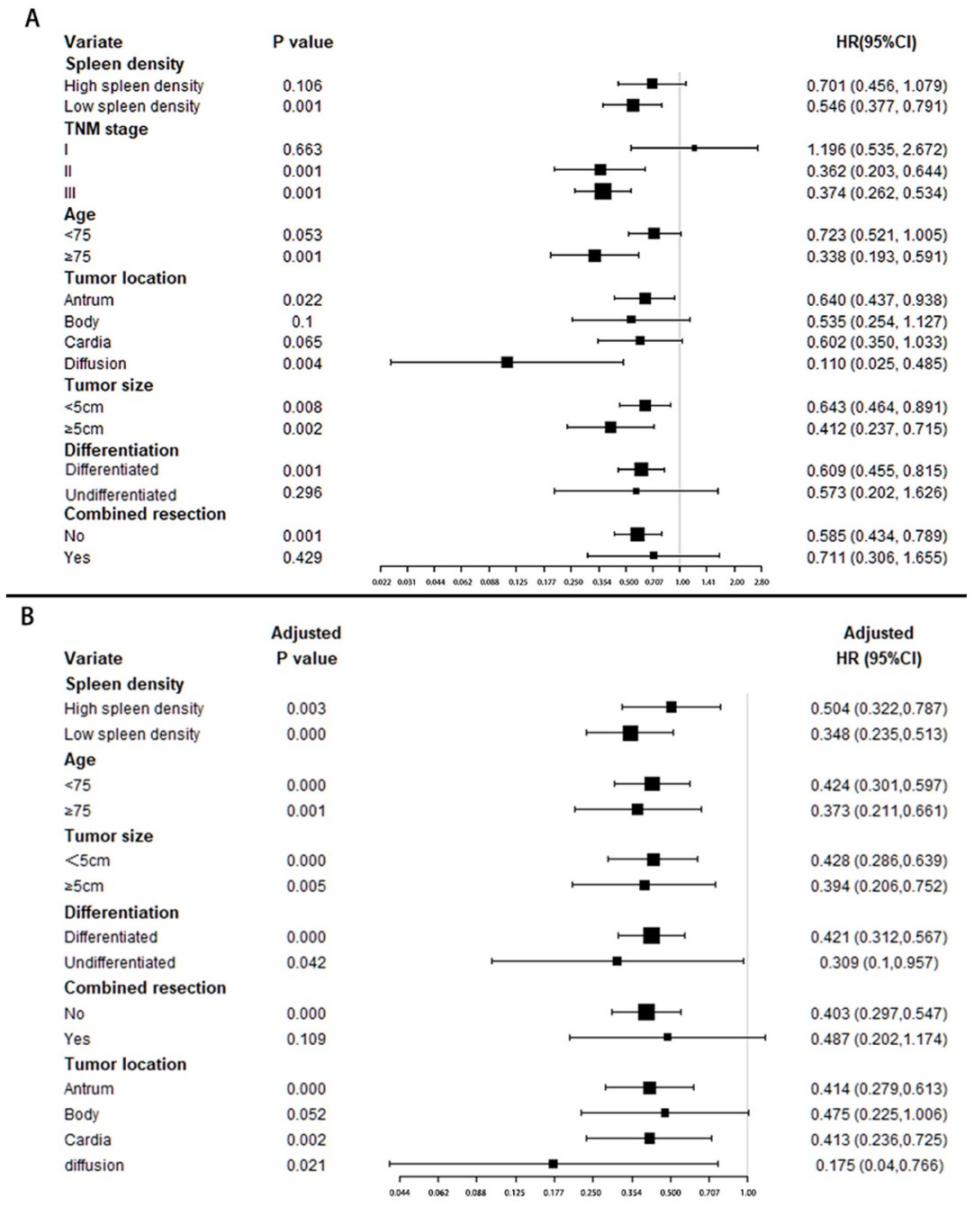
** Stratified analysis of adjuvant chemotherapy benefit.** The forest plot shows the benefit of chemotherapy in patients with different characteristics; (A). Univariate cox regression was used in each group. (B). Multivariate cox regression (adjusted by TNM stage) were used for adjusted chemotherapy benefit.

Forest plot (Figure 3A) shows adjuvant chemotherapy provided a better protective effect for patients with low splenic density (HR: 0.546, p=0.001), but no statistical significance in high splenic density group (HR:0.701, p=0.106).

Multivariate adjusted analysis (Figure 3B) showed patients in all groups benefited from chemotherapy. And there may be more chemotherapy benefits in patients with low spleen density and diffusion location, while there was no significant difference among other stratification factors.

## Discussion

Owing to clinical heterogeneity, patients with GC had large variations in clinical outcomes even among those with the same pathological feature 14, 15. Thus, a multiple prognostic assessment is essential for treatment decisions. The prediction of prognosis and chemotherapy benefits was improved by dividing patients with GC into high- or low-splenic density groups, with large differences seen in 3-year OS and DFS.

In the present study, splenic density varied at different tumor stages. Patients with larger tumor sizes, more metastasized LNs, and deeper tumor invasions showed lower splenic densities. Patients with low splenic densities tended to have poor 3-year DFS and OS rates (high vs low splenic density: 3-year DFS, 63.4% vs 44.6%, p < 0.001; 3-year OS, 69.8% vs 52.4%).

Cox regression analysis revealed that tumor size, location, differentiation, TNM stage, reconstruction method, age, ASA score, and adjuvant chemotherapy were the available prognostic indicators for patients with GC and have been reported by previous studies 16-18. Splenic density shows a high correlation with TNM stage and other prognostic factors, which may explain why splenic density was not a statistically significant prognostic indicator in multivariate analysis. However, splenic density is still an excellent indicator, especially in preoperative evaluation without accurate TNM stage.

Han et al. first established a murine orthotopic hepatocellular carcinoma (HCC) model and observed that the spleen significantly enlarged as the HCC progressed 13. Monitoring the splenocytes revealed that the proportion of almost all known immune cells (T cells, B cells, natural killer [NK] cells, and macrophages) decreased along with tumor progression 13. Changes in immune cells may explain the variable splenic density among the different tumor stages and the value of prognostic prediction. We first evaluated the relationship between splenic density and tumor characteristic in GCs, and the prognostic analysis among spleen densities proved its significance in prognosis prediction.

Adjuvant chemotherapy was recommended as a standard therapeutic strategy for advanced GC by the current guidelines; however, existing studies have shown that a GC subgroup does not benefit from chemotherapy 19-21. Thus, the identification of subgroups with different chemotherapy benefits will lead to a more personalized therapy and improve the prognosis of GC. The study by Chang et al. assessed the chemotherapy response at different ages and found no significance differences between the elderly and non-elderly 19. Yoon et al. indicated that tumor features extracted through CT radiomics helped discriminate HER2-positive GC patients who had better survival rates and received trastuzumab-based treatment 22. Radiomics is a recently proposed accurate prognosis prediction tool, but the complexity of feature extraction limits its unity in different centers 23. The present study indicates that chemotherapy provided better survival benefits to patients with low splenic densities and diffusion tumor location. To improve the treatment outcomes in patients with low splenic densities, more aggressive chemotherapy strategies may be identified at diagnosis. Therefore, splenic density is both a prognostic and predictive tool for GC patients. The mechanism of the relationship between splenic density and chemotherapy benefits was not thoroughly assessed. Trip et al. reported a radiation-induced progressive spleen volume decrease that led to pneumonia and fatal sepsis, possibly as a result of functional asplenia 24. Wen et al. 25 explored the variation of spleen volume in locally advanced non-small cell lung cancers during platinum-based chemotherapy, which confirmed the relationship between spleen functional variations and chemoradiotherapy. Low splenic density patients in the present study showed a more advanced tumor stage which may explain the relationship between splenic density and chemotherapy benefits. Owing to the huge inter-individual differences in spleen volume, the preoperative spleen volume was not statistically significant in prognosis analysis (data not shown). Compared with splenic volume, splenic density is a more sensitive and acceptable biomarker during tumor progression.

The present study was limited by the sample size and absence of posttreatment splenic features. It is unknown whether splenic density and volume change after radical gastrectomy. Furthermore, as the decision for adjuvant chemotherapy was not taken after a randomized comparison, treatment bias could not be eliminated. Therefore, a prospective study involving multiple centers is required to validate these results.

In summary, splenic density can be regarded as a new indicator of chemotherapy benefits and add prognostic value to the TNM staging system. Moreover, preoperative splenic evaluation may be a potential tool to guide individual treatment.

## Figures and Tables

**Table 1:** Clinical characteristics of patients according to splenic density

Factors	High splenic density (202)	Low splenic density (206)	p
Age, y	61.1 ± 11.5	65.9 ± 10.8	<0.001*
Gender			0.390
Male	40 (19.8%)	48 (23.3%)	
Female	162 (80.2%)	158 (76.7%)	
BMI, kg/m2	22.0 ± 3.0	21.7 ± 3.0	0.407
Platelet, 10^9^/L	207.0 ± 75.0	241.1 ± 86.5	<0.001
ASA grade			0.168
1	18 (8.9%)	9 (4.4%)	
2	159 (78.7%)	173 (84.0%)	
3	25 (12.4%)	24 (11.7%)	
Diabetes			0.403
No	186 (92.1%)	194 (94.2%)	
Yes	16 (7.9%)	12 (5.8%)	
Hypertension			0.925
No	164 (81.2%)	168 (81.6%)	
Yes	38 (18.8%)	38 (18.4%)	
Abdominal surgery history			0.508
No	173 (85.6%)	181 (87.9%)	
Yes	29 (14.4%)	25 (12.1%)	
Lymph node metastasis			<0.001*
N0	109 (54.0%)	61 (29.6%)	
N1	40 (19.8%)	42 (20.4%)	
N2	36 (17.8%)	54 (26.2%)	
N3	17 ( 8.4%)	49 (23.8%)	
Depth of invasion			0.003*
Tis/T1	58 (28.7%)	36 (17.5%)	
T2	27 (13.4%)	22 (10.7%)	
T3	61 (30.2%)	57 (27.7%)	
T4	56 (27.7%)	91 (44.2%)	
TNM stage			<0.001*
1	74 (36.6%)	41 (19.9%)	
2	65 (32.2%)	56 (27.2%)	
3	63 (31.2%)	109 (52.9%)	
Tumor size	3.4 ± 1.8	4.3 ± 2.1	<0.001*
Tumor location			0.340
Antrum	123 (60.9%)	127 (61.7%)	
Body	35 (17.3%)	24 (11.7%)	
Cardia	36 (17.8%)	46 (22.3%)	
diffusion	8 (4.0%)	9 (4.4%)	
Differentiation			0.219
Differentiated	185 (91.6%)	195 (94.7%)	
Undifferentiated	17 (8.4%)	11 (5.3%)	
Adjuvant chemotherapy			0.431
No	100 (49.5%)	110 (53.4%)	
Yes	102 (50.5%)	96 (46.6%)	
Combined resection			0.071
No	179 (88.6%)	193 (93.7%)	
Yes	23 (11.4%)	13 (6.3%)	
Reconstruction method			0.293
ROUX&Y	68 (33.7%)	83 (40.3%)	
Billroth I	55 (27.2%)	43 (20.9%)	
Billroth II	71 (35.1%)	75 (36.4%)	
Other	8 (4.0%)	5 (2.4%)	
Postoperative complication			0.211
No	145 (71.8%)	159 (77.2%)	
Yes	57 (28.2%)	47 (22.8%)	
Postoperative hospital stay, days	14.3 ± 15.7	13.2 ± 8.3	0.383
3-year RFS rate	128 (63.4%)	96 (46.6%)	<0.001*
3-year OS rate	141 (69.8%)	108 (52.4%)	<0.001*

Numbers given are mean+SD / N(%). † BMI, Body mass index; ASA, American Society of Anaesthesiologists; TNM, Tumor-lymph node-metastasis; RFS, Recurrence-free survival; OS, Overall survival * Statistically significant (P< 0.05)

**Table 2:** Univariable associations of the clinicopathological characteristics with disease-free survival and overall survival

Variables	Disease-free survival			Overall survival		
	Univariate HR (95%CI)	Univariate p value	Adjusted p value	Univariate HR (95%CI)	Univariate p value	Adjusted p value
Low splenic density	1.56 (1.20-2.04)	0.001*	0.477	1.62 (1.23-2.15)	<0.001*	0.500
Age >75	2.03 (1.51-2.73)	<0.001*	0.234	2.14 (1.57-2.91)	<0.001*	0.076
ASA grade >2	1.14 (0.77-1.70)	0.519		1.19(0.80-1.79)	0.395	
Diabetes	1.09 (0.65-1.81)	0.750		1.24 (0.74-2.06)	0.414	
Hypertension	1.20 (0.87-1.67)	0.273		1.09 (0.77-1.55)	0.619	
Abdominal surgery history	0.84 (0.56-1.27)	0.405		0.76 (0.49-1.19)	0.236	
TNM stage		<0.001*	<0.001*		<0.001*	<0.001*
1	Ref	Ref	Ref	Reference	Ref	Ref
2	2.71 (1.73-4.24)	<0.001	<0.001	2.32 (1.44-3.75)	0.001	<0.001
3	5.67 (3.76-8.55)	<0.001	<0.001	5.69 (3.70-8.77)	<0.001	<0.001
Tumor size>5cm	1.67 (1.23-2.26)	0.001*	0.801	1.62 (1.18-2.22)	0.003*	0.753
Tumor location		0.018*	0.158		0.021*	0.211
Antrum	Ref	Ref	Ref	Ref	Ref	Ref
Body	1.13 (0.77-1.67)	0.538	0.041	1.17 (0.78-1.76)	0.439	0.079
Cardia	1.63 (1.18-2.23)	0.003	0.085	1.67 (1.20-2.32)	0.002	0.092
diffusion	1.60 (0.86-2.96)	0.138	0.241	1.40 (0.71-2.76)	0.332	0.250
Differentiation		0.476			0.460	
Differentiated	Ref	Ref		Ref	Ref	
Undifferentiated	1.20 (0.72-2.00)	0.476		1.22 (0.72-2.06)	0.460	
Adjuvant chemotherapy	0.68 (0.52 -0.89)	0.004*	<0.001*	0.61(0.46 -0.80)	<0.001*	<0.001*
Combined resection	1.54 (1.02-2.31)	0.040*	0.033*	1.35 (0.87-2.10)	0.186	
Reconstruction method		<0.001*	0.001*		<0.001*	<0.001*
Roux&Y	Ref	Ref	Ref	Ref	Ref	Ref
Billroth I	0.37 (0.25-0.54)	<0.001	0.002	0.35 (0.24-0.53)	<0.001	0.002
Billroth II	0.56 (0.41-0.76)	0.002	<0.001	0.54 (0.39-0.74)	<0.001	<0.001
Other	0.96 (0.48-1.89)	0.899	0.437	1.06 (0.53-2.09)	0.877	0.292
Postoperative complication	1.05 (0.78-1.42)	0.756		1.10 (0.81-1.50)	0.542	

† HR, Hazard ratio; ASA, American Society of Anaesthesiologists; TNM, Tumor-lymph node-metastasis * Variate with statistically significant (P< 0.05) were put into multivariate analysis for adjusted p value.
